# Use of a Cybex NORM dynamometer to assess muscle function in patients with thoracic cancer

**DOI:** 10.1186/1472-684X-7-3

**Published:** 2008-04-10

**Authors:** Andrew Wilcock, Matthew Maddocks, Mary Lewis, Paul Howard, Jacky Frisby, Sarah Bell, Bisharat El Khoury, Cathann Manderson, Helen Evans, Simon Mockett

**Affiliations:** 1 Hayward House Macmillan Specialist Palliative Cancer Care Unit, Nottingham University Hospitals NHS Trust, Nottingham, UK; 2 Division of Physiotherapy Education, University of Nottingham, Nottingham, UK

## Abstract

**Background:**

The cachexia-anorexia syndrome impacts on patients' physical independence and quality of life. New treatments are required and need to be evaluated using acceptable and reliable outcome measures, e.g. the assessment of muscle function. The aims of this study were to: (i) examine the acceptability and reliability of the Cybex NORM dynamometer to assess muscle function in people with non-small cell lung cancer or mesothelioma; (ii) compare muscle function in this group with healthy volunteers and; (iii) explore changes in muscle function over one month.

**Methods:**

The test consisted of 25 repetitions of isokinetic knee flexion and extension at maximal effort while seated on a Cybex NORM dynamometer. Strength and endurance for the quadriceps and hamstrings were assessed as peak torque and total work and an endurance ratio respectively. Thirteen patients and 26 volunteers completed the test on three separate visits. Acceptability was assessed by questionnaire, reliability by intraclass correlation coefficients (ICC) and tests of difference compared outcomes between and within groups.

**Results:**

All subjects found the test acceptable. Peak torque and work done were reliable measures (ICC >0.80), but the endurance ratio was not. Muscle function did not differ significantly between the patient and a matched volunteer group or in either group when repeated after one month.

**Conclusion:**

For patients with non-small cell lung cancer or mesothelioma, the Cybex NORM dynamometer provides an acceptable and reliable method of assessing muscle strength and work done. Muscle function appears to be relatively well preserved in this group and it appears feasible to explore interventions which aim to maintain or even improve this.

## Background

The cachexia-anorexia syndrome is common in people with thoracic cancer impacting upon their activities of daily living, physical independence and quality of life [1,2]. Generally, treatment is reactive, when symptoms are well established, e.g. corticosteroids and progestins, but these have a catabolic effect which may accelerate the decline in muscle function [3,4]. New treatments are required.

Our group is working to develop proactive interventions such as exercise therapies which could be offered to patients soon after diagnosis, when they are most likely to have a reasonable performance status and limited weight loss, with the aim of preserving physical independence for as long as possible. These interventions would have a particular focus on maintaining the performance of the main muscles of ambulation, i.e. the quadriceps and hamstrings. Evaluation of these interventions requires an acceptable and reliable method of assessing leg muscle function, particularly endurance, as this is probably of greater functional relevance than strength [5,6]. The Cybex NORM dynamometer (Cybex, division of Lumex, Inc., Ronkonkoma, New York, USA) has been used to assess leg muscle function in healthy volunteers and various patient groups, but it has not been widely used in people with cancer, particularly thoracic cancer [7-9]. Thus, we have examined the acceptability and reliability of the Cybex NORM dynamometer to assess aspects of muscle function in people with non-small cell lung cancer or mesothelioma, a good performance status and limited weight loss. To examine for any early evidence of muscle function impairment in patients despite a reasonable performance status, we also studied a group of healthy volunteers, matched for sex, age and physical activity behaviour. To begin to explore the rate of decline in muscle function we repeated the test after one month.

## Methods

### Subjects

Patients with non-small cell lung cancer or mesothelioma and an Eastern Co-operative Oncology Group (ECOG) performance status of 0 or 1 were recruited from respiratory or oncology outpatient clinics. Patients were ineligible if they had weight loss ≥ 10% of their normal body weight, pain or pathology preventing leg exercise (e.g. arthritis, peripheral vascular disease, ischaemic heart disease), chronic obstructive pulmonary disease, radio- or chemotherapy within the previous month or medication changes within the previous week. Healthy volunteers were recruited from the staff and volunteers at Hayward House. All subjects gave written informed consent and Nottingham City Hospital Ethics Committee approved the study (ref. EC02/123).

### Measurements

#### Acceptability

Assessed following the first two visits by a questionnaire which asked if the subject would be prepared to repeat the test and also invited open comments about the test. If subjects indicated that they were not prepared to repeat the test or provided negative comments, further details were requested.

#### Physical activity behaviour

Assessed by the 12-item Modified Baecke Questionnaire which summarizes habitual physical household, sporting and leisure activities in the last year. Scoring takes into account the net energy cost of the activity and a higher score indicates a higher level of activity. A mean (range) score of 14 (1–31) has been reported in healthy older adults [10].

#### Muscle function

**Figure 1:**
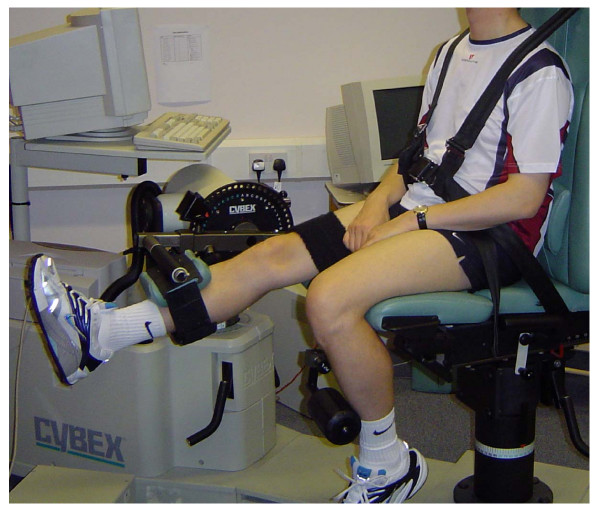
**Cybex NORM dynamometer set up**. The anatomical axis of rotation of the knee joint was aligned with the machine's axis of rotation and safety stops placed at the extremes of extension and flexion.

Assessed using a Cybex NORM dynamometer situated in the Human Performance Laboratory at City Hospital Campus, Nottingham University Hospitals NHS Trust. Subjects were seated in a chair with a lumbar back support and straps at the level of the shoulders, pelvis and thighs to minimize unwanted movement. The padded lever arm of the dynamometer was placed on the shin of the dominant leg, i.e. the one the subject would kick a ball with, and attached with straps. The contra-lateral limb was stabilized with a padded support. The seat was adjusted so that the anatomical axis of rotation of the knee joint was aligned with the axis of rotation of the dynamometer. The maximal range of movement at the knee joint was set with safety stops placed at the extremes of extension and flexion (Figure 1).

Equipment set up and test settings were standardized for each subject during all test visits. The dynamometer was set up to only allow isokinetic exercise, i.e. for any force exerted the machine produced an equivalent resistance and thus the lower leg could only move at a prefixed speed. The following measures of muscle function were obtained using the data capture and analysis software (version 2.06) provided with the dynamometer:

Strength – peak torque (Newtons, N) during 25 repetitions of flexion and extension was used to indicate the maximum strength of the hamstrings and quadriceps respectively.

Endurance – assessed using absolute and relative parameters:

Absolute – the sum of work done for all 25 repetitions. Work, expressed in Joules (J), equals torque (N) multiplied by the angular displacement (radians) and represents the area under the torque curve for each direction of movement for all repetitions [11].

Relative – work performed in the last 5 repetitions was divided by the work performed in the first 5 repetitions and expressed as a percentage to obtain an endurance ratio. This provides an indication of the subject's ability to maintain the initial workload [11].

### Protocol

Subjects attended on three separate days, 48 h and one month apart. Each visit was at the same time of day and lasted 30 min. Subjects were asked to avoid caffeine drinks within one hour, large meals within two hours and excess alcohol the night before the tests. Each test began with a warm up period of 10 min walking at a normal pace. While seated in the Cybex, subjects performed five sub-maximal extensions and flexions for familiarization purposes [12]. Subjects then performed 25 repetitions at 180° sec^-1 ^at maximal effort; this is considered sufficient exercise to assess endurance [12,13]. Standardized verbal encouragement was given to all subjects by the same researcher (CM). No visual feedback or advice on technique was given.

### Analysis

Data were assessed for normality and expressed as mean and standard-deviation or median and interquartile range as appropriate. Data from the first and second visits for patients and all volunteers were combined and used to assess between day reliability of muscle function outcomes, expressed as the standard deviation of the difference between tests and the intraclass correlation coefficient with 95% confidence intervals (values <0.6 indicate poor reliability) [14]. To examine for differences between patients and volunteers, muscle function outcomes achieved on the first visit were compared between patients and a subgroup of volunteers, matched as closely as possible for age, sex and Modified Baecke Score, using either the Student's t-test or the Wilcoxon signed-rank test as appropriate. To examine for change in muscle function over one month, subject outcomes for the second and third visits were similarly compared. Calculations were performed using Statistical Package for the Social Sciences (SPSS) version 14.0. A p value of <0.05 was regarded as statistically significant.

## Results

Sixteen patients and 28 healthy volunteers were recruited. Two patients and two volunteers did not complete the study due to unexpected deterioration or illness respectively in the intervening month; one patient's data was lost because of technical failure (Table 1). Eight and five patients respectively had a diagnosis of non-small cell lung cancer or mesothelioma, local (stage II (3)/III (5)) or metastatic disease, and a performance status of 0 or 1. Six patients had received palliative chemotherapy, five radio- and chemotherapy and one radiotherapy. None had undergone surgical resection. Median (range) body mass index was 27 (19–31) kg/m^2 ^and percentage weight loss was 1 (-7–5) %. To date, 12 have died with a median survival of 51 (range 10–114) weeks.

**Table 1:** Patient and volunteer details.

	*Patients*	*Matched volunteers*	*All volunteers*
*Number*	13	13	26
*Sex (M:F)*	6:7	6:7	10:16
*Mean (SD) age*	63 (6)	63 (6)	59 (9)
*Mean (SD) Modified Baecke Questionnaire*	12 (7)	11 (5)	13 (11)

### Acceptability

All subjects reported that they found the test acceptable and completed all tests. Two subjects reported minor discomfort from the strap around the leg (one patient) or the contra-lateral limb stabilizer (one volunteer). All subjects indicated that they would be prepared to complete the test again.

### Reliability

Intraclass correlation coefficient values of >0.8 were obtained for peak torque (muscle strength) and work done (absolute parameter of endurance) in both the hamstrings and quadriceps muscles. The endurance ratios (relative parameter of endurance) were a less reliable measure with intraclass correlation coefficient values of <0.6 (Table 2).

**Table 2:** Between day reliability of peak torque, work done and endurance ratio in all patients and volunteers (n = 39).

	*Mean value for the tests*	*Mean difference between the tests*	*SD of the difference between tests*	*Intraclass correlation coefficient (95% CI)*
*Quadriceps*				
Peak torque (N)	69	9	14	0.91 (0.82–0.95)
Work (J)	1098	125	321	0.84 (0.69–0.92)
Endurance ratio (%)	87	-15	38	0.56 (0.17–0.77)
*Hamstrings*				
Peak torque (N)	42	8	13	0.86 (0.75–0.93)
Work (J)	682	110	197	0.88 (0.77–0.94)
Endurance ratio (%)	102	-14	60	0.49 (0.02–0.74)

SD = standard deviation; CI = confidence interval

### Difference in muscle function between patients and matched volunteers

Compared to volunteers, patients produced a lower peak torque, did less work and had a higher endurance ratio for both the quadriceps and hamstrings, but the differences were not significant (Table 3).

**Table 3:** Difference between peak torque, work done and endurance ratio in patients and matched volunteers. Values are median [inter-quartile range].

	*Patients (n = 13)*	*Matched volunteers (n = 13)*	*p value*
*Quadriceps*			
Peak torque (N)	65 [27–71]	74 [54–84]	ns
Work (J)	821 [576–1386]	1057 [960–1393]	ns
Endurance ratio (%)	84 [72–108]	75 [70–90]	ns
*Hamstrings*			
Peak torque (N)	32 [13–39]	48 [36–54]	ns
Work (J)	515 [167–706]	721 [540–902]	ns
Endurance ratio (%)	95 [91–164]	88 [79–120]	ns

### Difference in muscle function in patients and matched volunteers over time

After one month, peak torque, work done or endurance ratio for the quadriceps or hamstrings did not differ significantly in either the patient or matched volunteer group (Table 4).

**Table 4:** Change in peak torque, work done and endurance ratio in the quadriceps and hamstrings over one month. Values are mean (SD) except when given as median [inter-quartile range].

	*Visit 2*	*Visit 3*	*p value*
*Patients (n = 13)*			
*Quadriceps*			
Peak torque (N)	63 (26)	58 (27)	ns
Work (J)	916 (459)	973 (493)	ns
Endurance ratio (%)	73 [62–84]	77 [68–93]	ns
			
*Hamstrings*			
Peak torque (N)	41 (21)	30 (14)	ns
Work (J)	612 (280)	501 (279)	ns
Endurance ratio (%)	92 [79–103]	87 [83–99]	ns
			
*Matched volunteers (n = 13)*			
*Quadriceps*			
Peak torque (N)	73 (21)	75 (25)	ns
Work (J)	1262 (341)	1160 (370)	ns
Endurance ratio (%)	76 [69–93]	77 [68–91]	ns
			
*Hamstrings*			
Peak torque (N)	46 (26)	47 (18)	ns
Work (J)	752 (202)	708 (265)	ns
Endurance ratio (%)	85 [74–108]	86 [67–91]	ns

## Discussion

We have demonstrated that leg exercise on a Cybex NORM dynamometer, involving extension and flexion at the knee joint, is generally acceptable to patients with non-small cell lung cancer or mesothelioma and produces reliable measures of peak torque (strength) and work done but not of an endurance ratio. Others have also found absolute parameters more reliable than relative parameters when assessing muscle endurance [12,13,15].

Compared to age, sex and physical activity behaviour matched volunteers, there was a tendency for patients to produce lower values of peak torque and work. We could find no similar study to directly compare our findings to, but reduced levels of work have been found in patients with small cell lung cancer, all in remission, six weeks after completing chemoradiation therapy when compared to healthy volunteers [16]. Differences in cancer type, treatment received and methodology may all contribute to the variance in the findings. For example, a different exercise protocol was used and patients and volunteers do not appear to have been matched for physical activity behaviour [16]. We also found that the endurance ratio was higher in the patient group compared to the matched volunteers. One possible explanation is that despite standardized encouragement, patients did not work as hard in the first five compared to the last five repetitions and thus appear to have fatigued less than the volunteers. Alternatively, it may reflect the unreliability of the endurance ratio. However, none of the differences in peak torque, work done or the endurance ratio were statistically significant. This suggests that muscle function is relatively well preserved in patients with non-small cell lung cancer or mesothelioma and a good performance status.

There was a general tendency for the patient group to get weaker over a period of four weeks, although the changes were not statistically significant. This period was selected because it reflects the time generally required for benefit to become apparent with the types of intervention we wish to pursue in the future, e.g. exercise training. Longitudinal studies of longer duration are required to identify the usual rate of decline in this patient group and to help put into context any benefit achieved with such interventions. Our data can be used to calculate the sample sizes required for adequately powered studies. For example, a pilot study examining for benefit of an exercise therapy, would require a sample size of 36 patients to reliably detect a change of 20% in peak torque and work done (80% power, p = 0.05).

Disadvantages to the use of the Cybex Norm Dynamometer include its cost and limited availability. The form of exercise is also unfamiliar for patients and appropriate familiarization with the equipment is required in order to achieve a maximal performance [7,17].

We have continued to use the Cybex NORM dynamometer in this group of patients, and are currently exploring the clinical relevance of measures of peak torque and work done by comparing them with performance ability in the incremental and endurance shuttle walking tests and free-living physical activity levels, assessed by an activity monitor.

## Conclusion

Exercise on a Cybex NORM dynamometer is an acceptable and reliable method of measuring peak torque and work done in the quadriceps and hamstrings of patients with lung cancer or mesothelioma and a good performance status. Muscle function appears to be relatively well preserved in this group, and this provides a window of opportunity for interventions designed to maintain or even improve muscle function, e.g. exercise training.

## Competing interests

The author(s) declare that they have no competing interests.

## Authors' contributions

AW conceived the study and supervised the research throughout, made substantial contributions to the analysis and interpretation of data and helped prepare the manuscript for publication. MM made substantial contributions to the analysis and interpretation of data, performed the statistical analysis and helped revise and prepare the manuscript for publication. ML, PH, JF, SB, CM, BeK and HE participated in the coordination of the study, made substantial contributions the acquisition of data and helped draft the manuscript. SM helped conceive the study and co-supervised the research throughout, made substantial contributions to the analysis and interpretation of data and helped draft the manuscript.

## Pre-publication history

The pre-publication history for this paper can be accessed here:


